# Supplementary material for: Using LC-MS/MS to Determine Salivary Steroid Reference Intervals in a European Older Adult Population
Source: Metabolites. 2023 Feb 13;13(2):265. doi: 10.3390/metabo13020265 (PMC9963097; doi:10.3390/metabo13020265)
Supplement: Supplementary file 1 [file metabolites-13-00265-s001.zip › metabolites-2174221-supplementary.pdf]

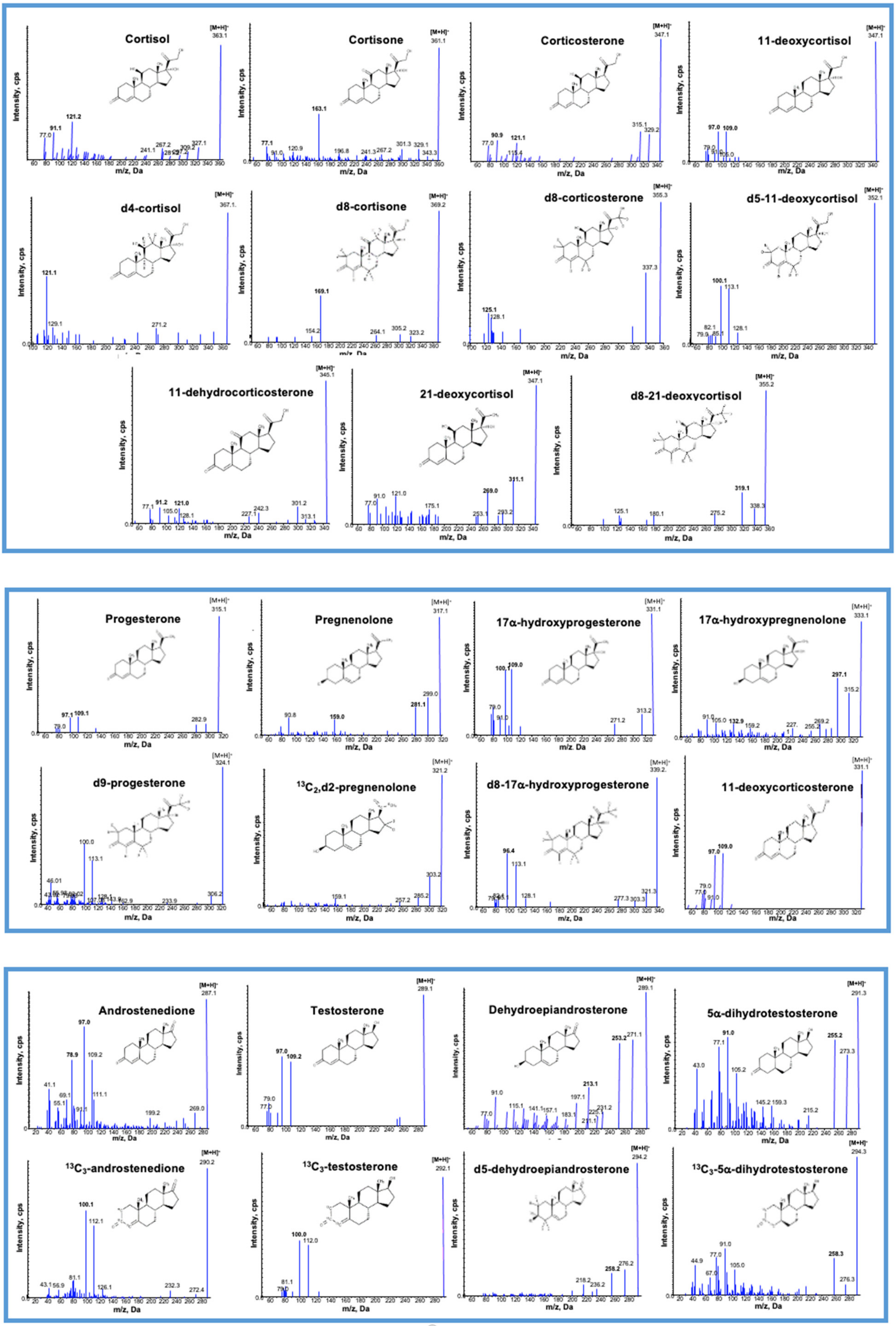

Figure S1 Electrospray ionisation product ion mass spectra of protonated **androgens, progestogens and corticoids** prepared in 50% methanol at 10 ng/mL or less, through infusion at a flow rate of 2 mL/min into a QTrap 6500+ turbospray electrospray ionisation source in positive mode, with a collision energy offset of 46V. Precursor ion masses

are indicated on the right hand side of each mass spectrum and product ions selected for the method are indicated in bold.

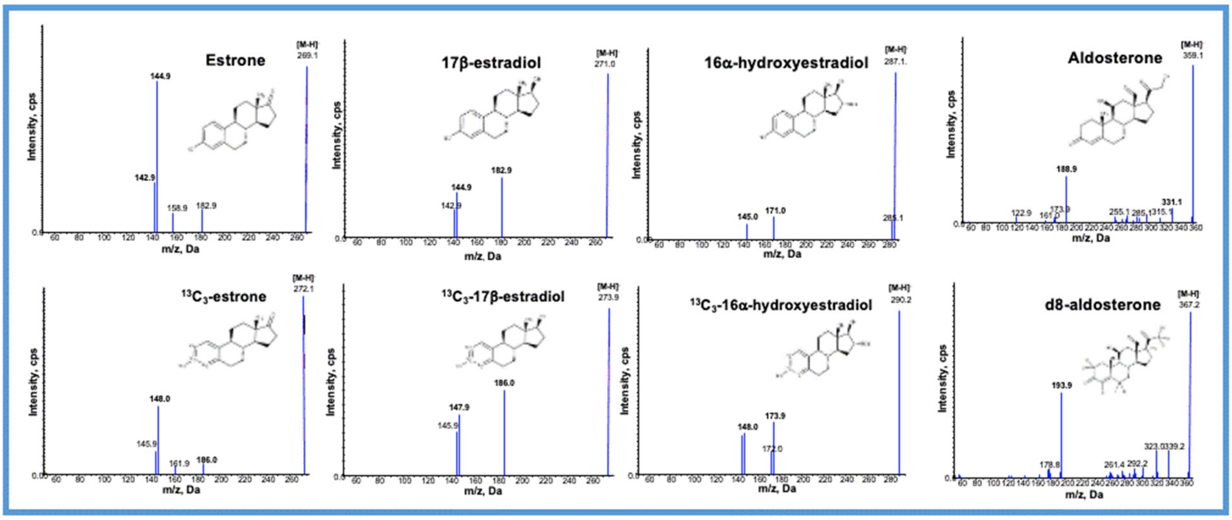

Figure S2 Electrospray ionisation product ion mass spectra of deprotonated estrogens and aldosterone prepared in 50% methanol at 1 mg/mL or less, through infusion at a flow rate of 2 mL/min into a QTrap 6500+ turbospray electrospray ionisation source in negative mode, with a collision energy offset of 46V. Precursor ions for estrone, estradiol, estriol and aldosterone were *m/z* 269.1, 271.0, 290.1 and 359.1, respectively. Precursor ions for their isotopically labelled counterparts were *m/z* 272.1, 273.9, 290.2 and 367.2, respectively.

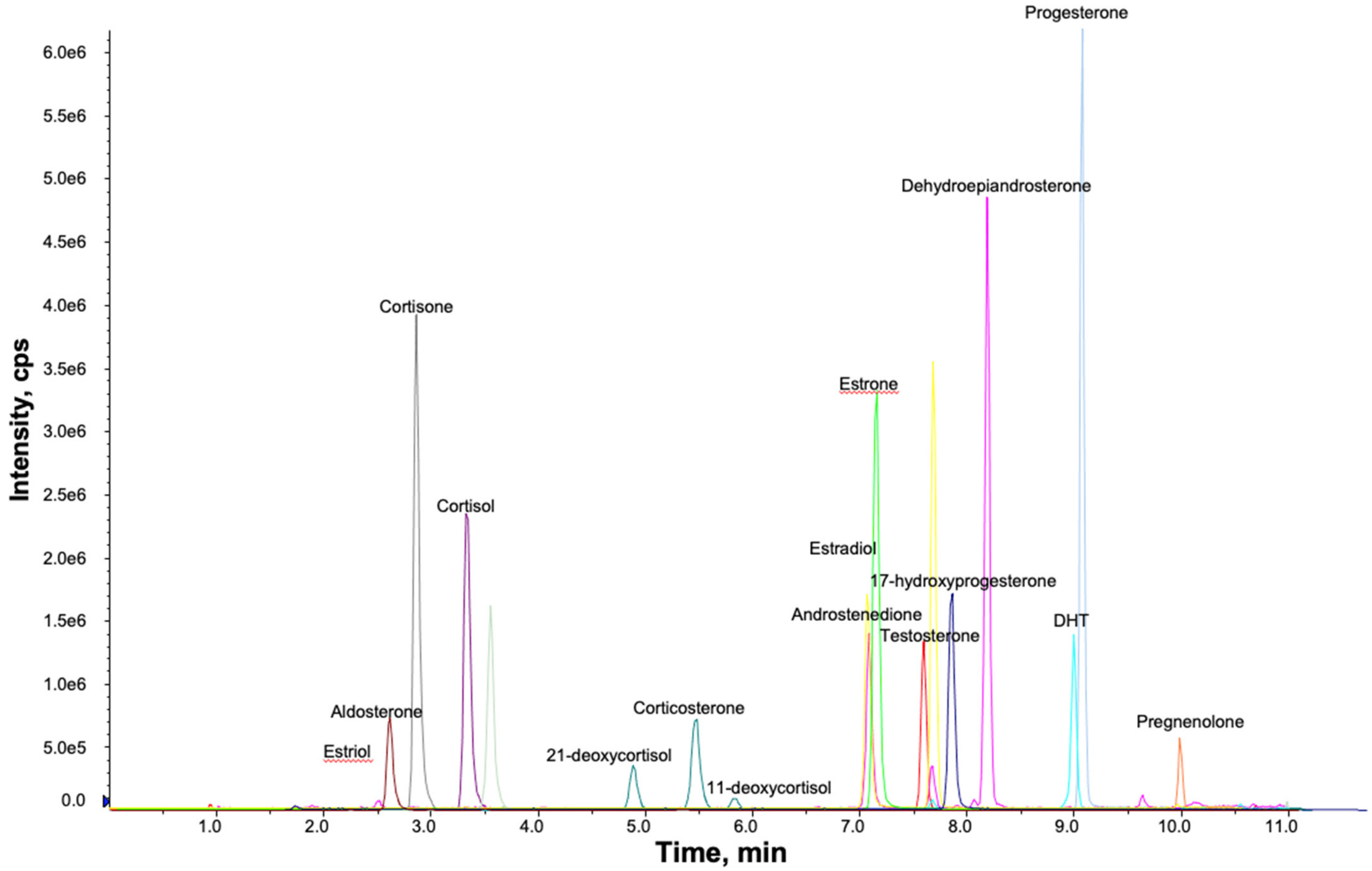

Figure S3. Representative chromatogram of steroids measured by LC-MS/MS, with quantifier parent-product ion transitions. Certified reference steroids ranging between 0.1 ng - 10 ng, separated on a Kinetex C18 (2.1 x 150 mm; 2. 6um) column. Positive ions for all steroids except estrone, estradiol, 16a-hydroxyestradiol and aldosterone.

Table S1. Confidence intervals (95%) of steroid hormones in full cohort, by sex and by disease status. All data presented in nM; AD: Alzheimer's disease; CI: confidence interval; n: number; A4- Androstenedione; T- Testosterone; DHEA- Dehydroepiandrosterone; DHT- 5α-dihydrotestosterone; P4- Progesterone; Preg- Pregnenolone; 17αOH-Preg- 17α-hydroxypregnenolone; 17αOH-P4- 17α-hydroxyprogesterone; 11-DOC- 11-deoxycorticosterone; A- 11-dehydrocorticosterone; S- 11-deoxycortisol; 21-DF- 21-deoxycortisol; B-corticosterone; E- cortisone; F- cortisol; . E1- Estrone; E2- Estradiol; E3- 16-hydroxyestradiol; Aldo- Aldosterone; nM: nanomolar.

Steroid	Sample timepoint	All		Male		Female		Healthy		AD biomarker positive	
		n	95% CI	n	95% CI	n	95% CI	n	95% CI	n	95% CI
A4	1 (~08:18am)	45	0.1203, 0.1777	28	0.1349, 0.2110	17	0.0769, 0.1355	26	0.1054, 0.1568	19	0.1216, 0.2243
	2 (~11:24am)	43	0.0962, 0.1327	28	0.1016, 0.1557	15	0.0712, 0.1015	30	0.0902, 0.1376	13	0.0855, 0.1513
	3 (~15:28pm)	35	0.0822, 0.1399	25	0.0836, 0.1572	10	0.0629, 0.1007	27	0.0756, 0.1224	8	0.0781, 0.2365
	4 (~21:43pm)	36	0.0828, 0.1441	21	0.0955, 0.1570	15	0.0456, 0.1604	25	0.0737, 0.1522	11	0.0756, 0.1745
T	1 (~08:18am)	60	0.0590, 0.0899	48	0.0694, 0.1032	12	0.0156, 0.0317	36	0.0661, 0.1072	24	0.0368, 0.0782
	2 (~11:24am)	61	0.0538, 0.0798	50	0.0634, 0.0919	11	0.0080, 0.0247	39	0.0538, 0.0862	22	0.0406, 0.0799
	3 (~15:28pm)	60	0.0457, 0.0699	48	0.0537, 0.0813	12	0.0063, 0.0272	36	0.0475, 0.0812	24	0.0320, 0.0660
	4	54	0.0481, 0.0738	46	0.0532, 0.0804	8	0.0069, 0.0293	34	0.0452, 0.0768	20	0.0309, 0.0809

	(~21:43pm)										
DHEA	1 (~08:18am)	56	0.5452, 1.0019	29	0.5164, 1.2895	27	0.4525, 0.7937	37	0.6494, 1.2824	19	0.2292, 0.5914
	2 (~11:24am)	59	0.4627, 0.9021	36	0.5379, 1.1470	23	0.2634, 0.5834	38	0.4933, 1.0735	21	0.3144, 0.6235
	3 (~15:28pm)	62	0.4326, 1.1447	33	0.4260, 1.6360	29	0.3165, 0.7302	40	0.4023, 1.4663	22	0.3564, 0.8098
	4 (~21:43pm)	64	0.3894, 0.7317	31	0.3323, 0.9074	33	0.3247, 0.6838	39	0.2932, 0.5595	25	0.3872, 1.1325
DHT	1 (~08:18am)	6	0.0858, 0.4147	4	0.0370, 0.5243	2	0.0551, 0.3013	6	0.0875, 0.4126	0	NA
	2 (~11:24am)	6	0.0347, 0.4698	4	0.0336, 0.6917	2	0.0034, 0.0930	6	0.0310, 0.4723	0	NA
	3 (~15:28pm)	6	0.1225, 0.9848	4	0.0430, 1.3972	2	0.1291, 0.5165	6	0.1269, 1.0230	0	NA
	4 (~21:43pm)	6	0.0772, 0.4807	4	0.1429, 0.2324	2	0.0215, 0.5802	6	0.0775, 0.4809	0	NA
P4	1 (~08:18am)	27	0.0319, 0.1178	14	0.0300, 0.1727	13	0.0187, 0.0996	20	0.0270, 0.1139	7	0.0161, 0.2116

	2 (~11:24am)	24	0.0396, 0.1355	11	0.0348, 0.1783	13	0.0125, 0.1536	16	0.0168, 0.0936	8	0.0517, 0.2764
	3 (~15:28pm)	27	0.0570, 0.2088	12	0.0296, 0.2800	15	0.0235, 0.2359	17	0.0194, 0.0610	10	0.1206, 0.4604
	4 (~21:43pm)	24	0.0619, 0.2595	15	0.0599, 0.3149	9	0.0179, 0.2721	15	0.0506, 0.3030	9	0.0190, 0.2993
Preg	1 (~08:18am)	28	0.1171, 0.2486	14	0.0998, 0.3036	14	0.0882, 0.2598	15	0.0521, 0.2214	13	0.1489, 0.3460
	2 (~11:24am)	28	0.1365, 0.3264	13	0.1158, 0.4934	15	0.1038, 0.2548	15	0.0824, 0.2617	13	0.1437, 0.4789
	3 (~15:28pm)	25	0.1321, 0.2454	11	0.0932, 0.2757	14	0.1199, 0.2472	13	0.0910, 0.2361	12	0.1346, 0.2845
	4 (~21:43pm)	28	0.1423, 0.2472	13	0.1579, 0.2924	15	0.0919, 0.2397	16	0.0941, 0.2216	12	0.1643, 0.3212
17αOH-Preg	1 (~08:18am)	4	0.0045, 0.2203	1	NA	3	0.0045, 0.2923	2	0.0557, 0.2923	2	NA
	2 (~11:24am)	7	0.1188, 0.4229	2	0.1053, 0.78073	5	0.1165, 0.4313	3	0.1053, 0.2256	4	0.0835, 0.6156
	3	4	0.0045, 0.0604	2	0.0045, 0.0790	2	0.0045, 0.0211	2	0.0045, 0.0790	2	0.0045, 0.0211

	(~15:28pm)										
	4 (~21:43pm)	4	0.0011, 0.1365	1	NA	3	0.0000, 0.1805	1	NA	3	0.0000, 0.1805
17αOH-P4	1 (~08:18am)	31	0.0537, 0.1164	18	0.0476, 0.1187	13	0.0368, 0.1435	19	0.0488, 0.1310	12	0.0287, 0.1194
	2 (~11:24am)	28	0.0528, 0.1189	18	0.0517, 0.1283	10	0.0266, 0.1346	17	0.0617, 0.1373	11	0.0219, 0.1064
	3 (~15:28pm)	20	0.0278, 0.0755	14	0.0325, 0.0870	6	0.0045, 0.0765	10	0.0263, 0.0985	10	0.0160, 0.0732
	4 (~21:43pm)	23	0.0363, 0.0952	15	0.0378, 0.1081	8	0.0043, 0.1100	18	0.0414, 0.1145	5	0.0042, 0.0469
11-DOC	1 (~08:18am)	10	0.0080, 0.0301	4	0.0053, 0.0416	6	0.0048, 0.0305	7	0.0082, 0.0370	3	0.0076, 0.0151
	2 (~11:24am)	8	0.0106, 0.0350	6	0.0095, 0.0335	2	0.0030, 0.0515	7	0.0091, 0.0370	1	NA
	3 (~15:28pm)	11	0.0107, 0.0341	5	0.0070, 0.0372	6	0.0055, 0.0376	9	0.0188, 0.0387	2	0.0030, 0.0076
	4 (~21:43pm)	10	0.0109, 0.0378	6	0.0108, 0.0436	4	0.0030, 0.0428	7	0.0158, 0.0471	3	0.0030, 0.0136

A	1 (~08:18am)	97	0.826, 1.214	50	0.6914, 1.0676	47	0.844, 1.549	59	0.7283, 1.2579	38	0.810, 1.385
	2 (~11:24am)	87	0.3223, 0.5402	43	0.3127, 0.7068	44	0.2861, 0.4225	53	0.2614, 0.3727	34	0.3514, 0.8211
	3 (~15:28pm)	76	0.2413, 0.3582	41	0.2335, 0.3751	35	0.2155, 0.3897	47	0.2181, 0.3572	29	0.2364, 0.4030
	4 (~21:43pm)	62	0.1151, 0.2454	33	0.0980, 0.2698	29	0.0931, 0.2813	38	0.1074, 0.2601	24	0.0831, 0.2978
S	1 (~08:18am)	58	0.611, 0.0937	28	0.0465, 0.0905	30	0.0642, 0.1070	36	0.0587, 0.0986	22	0.0533, 0.1007
	2 (~11:24am)	40	0.0196, 0.0537	23	0.0228, 0.0818	17	0.0199, 0.0202	20	0.0186, 0.0374	20	0.0146, 0.0780
	3 (~15:28pm)	17	0.0065, 0.0152	10	0.0058, 0.0167	7	0.0035, 0.0179	6	0.0087, 0.0178	11	0.0033, 0.0156
	4 (~21:43pm)	5	0.0029, 0.0234	3	0.0029, 0.0289	2	0.0029, 0.0274	4	0.0029, 0.0281	1	NA
21-DF	1 (~08:18am)	2	0.700, 0.0734	2	0.700, 0.0734	0	NA	2	0.700, 0.0734	0	NA
	2	4	0.0058, 0.0318	3	0.0029, 0.0348	1	NA	2	0.0144, 0.0348	2	0.0029, 0.0289

	(~11:24am)										
	3 (~15:28pm)	6	0.0060, 0.0332	4	0.0011, 0.0325	2	0.0087, 0.0374	3	0.0000, 0.0374	3	0.0043, 0.0419
	4 (~21:43pm)	2	0.0058, 0.0447	1	NA	1	NA	2	0.0058, 0.0447	0	NA
B	1 (~08:18am)	27	0.0891, 0.2250	17	0.0941, 0.2825	10	0.0383, 0.2161	16	0.0456, 0.1571	11	0.1158, 0.3811
	2 (~11:24am)	9	0.0146, 0.6669	6	0.0154, 0.9815	3	0.0043, 0.0723	7	0.0074, 0.0417	2	0.0532, 1.9313
	3 (~15:28pm)	9	0.0130, 0.0410	7	0.0135, 0.0468	2	0.0072, 0.0159	7	0.0130, 0.0421	2	0.0029, 0.0548
	4 (~21:43pm)	8	0.0097, 0.0729	4	0.0130, 0.0245	4	0.0036, 0.1268	7	0.0122, 0.0824	1	NA
E	1 (~08:18am)	115	18.02, 23.58	59	16.09, 23.91	56	18.29, 26.20	70	17.59, 23.80	45	16.63, 26.51
	2 (~11:24am)	114	11.96, 16.78	60	12.05, 20.15	54	10.38, 14.73	69	10.40, 14.70	45	12.53, 22.20
	3 (~15:28pm)	113	8.094, 10.939	58	8.72, 13.22	55	6.579, 9.889	68	7.517, 10.813	45	7.590, 12.844

	4 (~21:43pm)	112	4.144, 6.101	59	4.215, 7.600	53	3.422, 5.499	66	4.062, 6.356	46	3.454, 7.151
F	1 (~08:18am)	114	4.851, 7.009	59	4.376, 7.605	55	4.649, 7.503	69	4.433, 7.008	45	4.436, 8.084
	2 (~11:24am)	110	2.243, 3.514	59	2.234, 4.356	51	1.903, 2.930	66	1.846, 2.870	44	2.375, 5.132
	3 (~15:28pm)	109	1.303, 2.094	57	1.341, 2.759	52	1.008, 1.534	65	1.129, 1.756	44	1.292, 2.896
	4 (~21:43pm)	104	0.809, 1.422	52	0.719, 1.559	52	0.687, 1.582	61	0.738, 1.444	43	0.698, 1.748
E1	1 (~08:18am)	25	0.0070, 0.0341	17	0.0045, 0.0126	8	0.0069, 0.0842	14	0.0099, 0.0532	11	0.0020, 0.0076
	2 (~11:24am)	22	0.0052, 0.0139	13	0.0045, 0.0118	9	0.0025, 0.0213	21	0.0054, 0.0149	1	NA
	3 (~15:28pm)	25	0.0046, 0.0144	16	0.0035, 0.0113	9	0.0015, 0.0247	17	0.0057, 0.0194	8	0.0016, 0.0050
	4 (~21:43pm)	19	0.0048, 0.0101	14	0.0034, 0.0148	5	0.0040, 0.0100	15	0.0057, 0.0118	4	0.0018, 0.0040
E2	1	12	0.0052, 0.0573	3	NA	9	0.0057, 0.0751	11	0.0054, 0.0621	1	NA

	(~08:18am)										
	2 (~11:24am)	9	0.0031, 0.0250	5	0.0029, 0.0295	4	0.0009, 0.0344	5	0.0015, 0.0282	4	0.0028, 0.0315
	3 (~15:28pm)	6	0.0037, 0.0315	4	0.0018, 0.0318	2	0.0037, 0.0445	3	0.0000, 0.0411	3	0.0037, 0.0445
	4 (~21:43pm)	6	0.0031, 0.0243	2	0.0055, 0.0437	4	0.0023, 0.0050	2	0.0028, 0.0337	4	0.0037, 0.0055
E3	1 (~08:18am)	12	0.0031, 0.0076	8	0.0044, 0.0093	4	0.0000, 0.0050	11	0.0039, 0.0081	1	NA
	2 (~11:24am)	11	0.0041, 0.0227	6	0.0042, 0.0162	5	0.0014, 0.0354	8	0.0030, 0.0277	3	0.0017, 0.0078
	3 (~15:28pm)	13	0.0020, 0.0054	8	0.0022, 0.0073	5	0.0007, 0.0035	10	0.0019, 0.0062	3	0.0000, 0.0045
	4 (~21:43pm)	9	0.0037, 0.1741	8	0.0031, 0.0073	1	NA	5	0.0047, 0.3095	4	0.0013, 0.0052
Aldo	1 (~08:18am)	16	0.0359, 0.0961	9	0.0236, 0.0673	7	0.0381, 0.1550	10	0.0279, 0.1167	6	0.0314, 0.0853
	2 (~11:24am)	20	0.0375, 0.0741	12	0.0226, 0.0603	8	0.0442, 0.1115	14	0.0350, 0.0793	6	0.0227, 0.0829

	3 (~15:28pm)	18	0.0298, 0.0794	10	0.0166, 0.0419	8	0.0373, 0.1363	15	0.0318, 0.0878	3	0.0000, 0.0895
	4 (~21:43pm)	14	0.0298, 0.0675	9	0.0252, 0.1068	5	0.0254, 0.0547	12	0.0302, 0.0712	2	0.0236, 0.0694

Table S2. Comparison of median values for steroids by sex and disease status. Data presented for all steroids where n>6. Comparisons analysed using Wilcoxon Signed Rank test in R. A4- Androstenedione; T- Testosterone; DHEA-Dehydroepiandrosterone; DHT- 5α-dihydrotestosterone; P4- Progesterone; Preg- Pregnenolone; 17αOH-Preg- 17α-hydroxypregnenolone; 17αOH-P4- 17α-hydroxyprogesterone; 11-DOC- 11-deoxycorticosterone; A- 11-dehydrocorticosterone; S-11-deoxycortisol; 21-DF- 21-deoxycortisol; B-corticosterone; E- cortisone; F- cortisol; . E1- Estrone; E2- Estradiol; E3- 16-hydroxyestradiol; Aldo- Aldosterone; W- Wilcoxon test statistic.

Steroid	Sample Time-point	Median		W	p	Median		W	p
		Male	Female			Healthy	AD		
A4	1	0.15	0.10	109.5	0.003	0.13	0.12	217	0.50
	2	0.11	0.09	151	0.13	0.10	0.11	180.5	0.71
	3	0.09	0.08	106	0.50	0.08	0.11	80	0.28
	4	0.10	0.05	83	0.02	0.08	0.10	112	0.39
T	1	0.08	0.02	117	0.002	0.07	0.04	551.5	0.07
	2	0.07	0.01	61.5	<0.001	0.06	0.07	472	0.52
	3	0.06	0.009	81	0.0001	0.06	0.04	512.5	0.23
	4	0.07	0.01	64	0.004	0.06	0.06	333.5	0.91
DHEA	1	0.39	0.60	400	0.90	0.68	0.16	502	0.009
	2	0.48	0.26	314	0.12	0.41	0.33	443	0.49
	3	0.56	0.23	422.5	0.43	0.41	0.46	432	0.91
	4	0.35	0.31	497.5	0.86	0.31	0.35	417	0.33
DHT	1	Insufficient sample size				Insufficient sample size			

	2	Insufficient sample size				Insufficient sample size			
	3	Insufficient sample size				Insufficient sample size			
	4	Insufficient sample size				Insufficient sample size			
P4	1	0.03	0.03	87	0.86	0.03	0.03	65	0.80
	2	0.03	0.02	46	0.14	0.02	0.03	43.5	0.22
	3	0.03	0.03	88	0.94	0.02	0.18	51.5	0.10
	4	0.03	0.03	61	0.72	0.03	0.03	68.5	0.98
Preg	1	0.17	0.17	90.5	0.74	0.005	0.20	66.5	0.15
	2	0.22	0.18	84	0.55	0.16	0.22	83	0.52
	3	0.20	0.18	76.5	1	0.17	0.21	63	0.43
	4	0.19	0.18	72	0.25	0.17	0.25	58	0.08
17αOH-Preg	1	Insufficient sample size				Insufficient sample size			
	2	0.44	0.15	4	0.86	0.15	0.21	5	0.86
	3	Insufficient sample size				Insufficient sample size			
	4	Insufficient sample size				Insufficient sample size			
17αOH-P4	1	0.06	0.05	120	0.92	0.06	0.04	134	0.43
	2	0.04	0.01	67	0.28	0.12	0.02	117.5	0.27
	3	0.03	0.008	23.5	0.14	0.02	0.02	61	0.43

	4	0.03	0.005	31.5	0.07	0.03	0.01	62	0.22
11-DOC	1	0.02	0.01	9	0.59	0.02	0.008	12	0.82
	2	0.02	0.03	7	0.86	0.02	0.02	4	1
	3	0.03	0.01	14	0.93	0.03	0.005	12.5	0.48
	4	0.03	0.008	10	0.74	0.03	0.003	17	0.16
A	1	0.74	0.71	1297	0.38	0.66	0.83	1059	0.65
	2	0.30	0.30	904.5	0.73	0.29	0.44	689	0.07
	3	0.22	0.19	660	0.55	0.22	0.21	607.5	0.43
	4	0.08	0.09	545.5	0.35	0.09	0.08	486.5	0.66
S	1	0.05	0.08	505	0.19	0.05	0.06	394.5	0.99
	2	0.03	0.01	125	0.06	0.02	0.02	230	0.42
	3	0.009	0.004	33	0.88	0.01	0.004	47.5	0.15
	4	Insufficient sample size				Insufficient sample size			
21-DF	1	Insufficient sample size				Insufficient sample size			
	2	Insufficient sample size				Insufficient sample size			
	3	Insufficient sample size				Insufficient sample size			
	4	Insufficient sample size				Insufficient sample size			
B	1	0.08	0.04	63	0.28	0.05	0.16	55	0.11

	2	0.03	0.01	6	0.55	0.99	0.01	1	0.11
	3	0.02	0.01	3.5	0.38	0.02	0.03	8	0.88
	4	0.02	0.009	5	0.49	0.02	0.003	7	0.25
E	1	19.18	19.09	1809	0.38	18.41	20.44	1565	0.96
	2	11.18	10.96	1494	0.48	9.62	13.17	1288	0.13
	3	9.24	5.44	1212.5	0.03	8.83	7.61	1450	0.64
	4	3.65	3.33	1420	0.40	3.75	3.33	1603	0.62
F	1	4.55	4.19	1722	0.57	4.30	4.22	1503.5	0.78
	2	2.17	1.89	1433.5	0.67	1.79	2.41	1165.5	0.08
	3	1.30	1.02	1249	0.16	1.05	1.47	1204.5	0.16
	4	0.53	0.62	1388	0.82	0.60	0.54	1334.5	0.88
E1	1	0.004	0.01	85.5	0.32	0.01	0.004	112.5	0.05
	2	0.004	0.004	46.5	0.44	0.004	0.002	17.5	0.30
	3	0.004	0.004	72	1	0.004	0.003	88	0.25
	4	0.004	0.004	38.5	0.78	0.005	0.003	46.5	0.10
E2	1	0.004	0.004	19.5	0.24	0.004	0.004	7.5	0.61
	2	0.004	0.004	9	0.90	0.004	0.01	8.5	0.80
	3	Insufficient sample size				Insufficient sample size			

	4	Insufficient sample size				Insufficient sample size			
E3	1	0.006	0.002	5.5	0.09	0.00	0.005	10.5	0.19
	2	0.008	0.003	9.5	0.36	0.006	0.008	14	0.76
	3	0.004	0.003	11	0.19	0.003	0.003	16.5	0.86
	4	0.005	0.51	8	0.17	0.009	0.004	15.5	0.22
Aldo	1	0.03	0.05	43	0.24	0.03	0.04	24.5	0.59
	2	0.03	0.04	72.5	0.06	0.04	0.03	52.5	0.41
	3	0.03	0.06	63	0.045	0.05	0.02	29	0.48
	4	0.03	0.06	33	0.18	0.04	0.05	10	0.78
